# Fluid restriction reduces pulmonary edema in a model of acute lung injury in mechanically ventilated rats

**DOI:** 10.1371/journal.pone.0210172

**Published:** 2019-01-17

**Authors:** Sarah A. Ingelse, Jenny Juschten, Martinus A. W. Maas, Gustavo Matute-Bello, Nicole P. Juffermans, Job B. M. van Woensel, Reinout A. Bem

**Affiliations:** 1 Pediatric Intensive Care Unit, Emma Children’s Hospital, Academic Medical Center, Amsterdam, The Netherlands; 2 Laboratory of Experimental Intensive Care and Anesthesiology (L·E·I·C·A), Academic Medical Center, Amsterdam, The Netherlands; 3 Department of Intensive Care, Academic Medical Center, Amsterdam, The Netherlands; 4 Department of Intensive Care and Research VUmc Intensive Care (REVIVE), VU Medical Center, Amsterdam, The Netherlands; 5 Center for Lung Biology, Division of Pulmonary, Critical Care and Sleep Medicine, University of Washington, and Medical Research Service, VA Puget Sound Healthcare System, Seattle, WA, United States of America; Ohio State University, UNITED STATES

## Abstract

Experimental acute lung injury models are often used to increase our knowledge on the acute respiratory distress syndrome (ARDS), however, existing animal models often do not take into account the impact of specific fluid strategies on the development of lung injury. In contrast, the current literature strongly suggests that fluid management strategies have a significant impact on clinical outcome of patients with ARDS. Thus, it is important to characterize the role of fluid management strategies in experimental models of lung injury. In this study we investigated the effect of two different fluid strategies on commonly used outcome variables in a short-term model of acute lung injury, in relation to age. Infant (2–3 weeks) and adult (3–4 months) Wistar rats received intratracheal instillations of lipopolysaccharide and 24 hours later were mechanically ventilated for 6 hours. During mechanical ventilation, rats from both age groups were randomized to either a standard or conservative intravenous fluid strategy. We found that the hemodynamic response in infant and adult rats was similar in both fluid strategies. Lung wet-to-dry ratios were lower in adult, but not in infant rats receiving the conservative fluid strategy as compared to the standard fluid strategy. There were age-related differences in markers of alveolar capillary barrier disruption and alveolar fluid clearance, yet these were unaffected by fluid strategy. Finally, we found significantly higher IL-1β and TNF-α concentrations in the adult rats treated with the conservative as compared to the standard fluid regimen. In conclusion, the choice of fluid strategy in mechanically ventilated rats with experimental LPS-induced acute lung injury has a significant effect on pulmonary extravascular water, an important and well-recognized lung injury marker, and on the local pro-inflammatory cytokine profiles. We advocate the use of a more uniform, conservative, fluid strategy regimen in experimental models of acute lung injury.

## Introduction

Over the last decades, accumulating evidence has shown that fluid overload is associated with adverse outcomes in critically ill patients requiring mechanical ventilation (MV). In a large clinical randomized controlled trial in adult ICU patients, a conservative fluid regimen was found to be associated with significantly more ventilator-free days and improved oxygenation [1]. The importance of fluid overload particularly holds true for patients with acute respiratory distress syndrome (ARDS) [2]. In patients with ARDS, early fluid overload, typically developing over the first 72 hours of admission, is associated with longer duration of MV and higher mortality (reviewed in [3, 4]). Therefore, conservative fluid management strategies, such as early intravenous fluid restriction, has become an important pillar of supportive treatment in many ICUs treating critically ill patients and patients with ARDS.

The likely underlying pathophysiological mechanism of the adverse effect of fluid overload is aggravation of extravascular and interstitial edema in the lungs and other organs [3], but may also include increased pro-inflammatory responses to fluid accumulation in the body [5, 6]. These negative effects appear to affect both adults and children with ARDS [2, 7, 8], although there are potential differences in the extent and consequences of fluid overload due to age-related changes in fluid homeostasis and total body-water distribution [3, 9].

The modelling of human ARDS by experimental induction of acute lung injury in animals has proven to be an important tool to increase our knowledge of this complex syndrome [10]. Given the above mentioned important influences of fluid treatment for the course of ARDS in humans it is remarkable that so far the potential effects of intravenous fluid maintenance strategies on markers of acute lung injury in animals have not been addressed. There is currently no protocol or consensus regarding a standard fluid strategy, resulting in the use of a wide range of fluid rates within models using the same animal species [9, 11]. Likewise, the potential effect of age-related differences in the response to fluids during acute lung injury remains unknown.

In general, experiments of acute lung injury in animals have a relatively short duration (hours) as compared to the clinical setting of human ARDS (days to weeks). Consequently, the impact of fluid overload, which is a relatively slow process, on clinical disease outcomes may be limited. However, we hypothesize that fluid strategy has potential impact on short-term and commonly used outcome variables in models of acute lung injury [12], such as measurements of lung edema and inflammatory responses. In addition, we hypothesize that this effect of fluid in acute lung injury may be dependent on age. To test this hypothesis, in this study we compare a standard to a conservative fluid maintenance strategy in a model of acute lung injury by a combination of LPS inoculation and mechanical ventilation in adult and infant rats. If our data supports our hypothesis, this may have important consequences for the design of experimental studies of acute lung injury in animals.

## Materials and methods

### Animal protocols

The animal protocols were approved by the national Ethical Committee for Animal Experiments (The Netherlands; protocol AVD118002016527). Animals were used in compliance with Institutional Standards for Use of Laboratory Animals. The rats were housed in a specific pathogen-free facility on a 12/12h light/dark cycle and 20–24°C ambient temperature and allowed to acclimatize for 7 days prior to start of the experiment. Infant (n = 16, 3–4 weeks, 68.8 ± 7.5 gr) and adult (n = 13, 3–4 months, 310.7 ± 62.7 gr) specific-pathogen free RccHan Wistar rats (male and female) received intratracheal inoculations of LPS, 5mg/kg (*Escherichia coli*, serotype: O127:B8, Sigma Aldrich, St. Louis, MO, USA) with a nebulizer (Penn-Century. Inc. Wyndmoor, PA microSprayer Aerosolizer–Model IA-1B or IA-1C-R) 24 hours before the onset of mechanical ventilation (MV).

At 24 hours after LPS inoculation, animals were anesthetized with 0.03 mg/kg buprenorphine (Temgesic; Indivior UK Limited, Slough, United Kingdom) subcutaneously and an intraperitoneal injection of pentobarbital sodium (80–85 mg/kg; Euthasol; AST farma BV, The Netherlands). Subsequently, animals received a tail vein cannula for administration of maintenance anesthesia and fluids. A tracheostomy was performed for initiation of MV. During MV, anesthesia was maintained by intravenous infusion of 20–30 mg/kg/hour pentobarbital sodium (Euthasol; AST farma BV, The Netherlands). A polyethylene catheter was inserted into the right carotid artery and connected to a pressure sensor in order to continuously monitor blood pressure and heart rate. Temperature was maintained at the normal body temperature for a rat (±37.5°C) using a heating pad.

The rats were mechanically ventilated in a pressure-controlled mode (Babylog 8000, Dräger, Germany) for 6 hours aiming at tidal volumes ranging from 6–8 ml/kg as measured by a pneumotachometer (HSE; Harvard Apparatus, Manheim, Germany) and recorded by respiration software (HSE-BDAS Basic Data Acquisition, Harvard Apparatus). Positive end-expiratory pressure (PEEP) was set at 3.5 cmH_2_O. The inspired oxygen fraction (FiO_2_) was set at 30% and the respiratory rate at 60 breaths per minute with an inspiratory/expiratory ratio of 1:1.5. The peak inspiratory pressure (PIP) was adjusted to maintain normocapnia (PaCO_2_ = 35–45 mmHg) and a normal pH (7.35–7.45). Static lung compliance was determined every hour upon an inspiratory hold.

Prior to the onset of MV, all rats were randomly allocated to one of the two experimental groups; standard fluid strategy or conservative fluid strategy (n = 6–8 for each group). These fluid strategies were based on pilot experiments measuring the blood pressure response to each strategy during 6 hours of MV. Volume associated with maintenance anesthesia was incorporated in the allocated fluid strategies. Ringer’s lactate solution was administered to reach the required volume of the allocated fluid strategy. The standard fluid regimen consisted of 6.5 ml/kg/hour intravenous fluid, based on the normal daily fluid intake of a healthy rat [13]. The conservative fluid regimen consisted of 2.5 ml/kg/hour intravenous fluid volume. Rats in the standard fluid regimen were pre-hydrated with an intravenous bolus of 10 ml/kg to mimic clinical resuscitative practice. If the mean arterial pressure decreased below <60 mmHg in adult animals or <40 mmHg in infant animals, the rats in the standard fluid strategy received a fluid bolus of 10 ml/kg, repeated every 10 min until blood pressure normalization. If hypotension persisted after three consecutive boluses a norepinephrine bolus (10 μg/kg) was given. Rats in the conservative fluid regimen received norepinephrine boluses without preceding fluid boluses.

The rats were euthanized after 6 hours of MV by intravenous injection of sodium pentobarbital and exsanguination. Bronchoalveolar lavage fluid (BALF) was obtained from the left lung by three separate aliquots of 0.9% NaCl (aliquot volume of 0.5 ml for infants and 2.0 ml for adults) and the aliquots were pooled. Immediately after performing the BAL, a fluid aliquot was retrieved for determination of total cell counts. The remainder of the bronchoalveolar lavage fluid (BALF) was centrifuged for 10 min at 1500 x *g*, 4°C and the supernatant stored at -80°C. The middle lobe of the right lung was flash-frozen for homogenization. The total water content in lung, kidney, liver and heart tissue was measured by calculating wet-to-dry weight ratios.

### Measurements

Inflammation was assessed by determining total cell counts of the BALF using a Burker hemacytometer (LO-Laboroptik Ltd, Lancing, UK); cytospin preparations were stained with Giemsa (Dade Behring AG, Dudingen, Switzerland) for determination of cell differentials. The concentrations of the pro-inflammatory cytokines IL-6, MIP-2, IL-10 and TNF-α in the BALF, and IL-1β in lung homogenate, were determined using the respective specific Quantikine ELISA Kits by R&D Systems (Abingdon, UK). Myeloperoxidase (MPO), an indicator of neutrophil activity, was analyzed in the lung homogenates and measured by the MPO Rat ELISA kit (HK105-02, Hycult Biotech, Sanbio B.V., Uden, The Netherlands). Disruption of the alveolar-capillary barrier was measured by determining protein extravasation into the alveolar spaces, as determined by the BALF concentrations of total protein and alpha-2-macroglobulin. Total protein was measured by the Lowry-protein determination. Alpha-2-macroglobulin was measured with the Rat Alpha-2-Macroglobulin ELISA (Life Diagnostics, Inc., MAC-2, West-Chester, USA). In addition, lung Na^+^/K^+^/ATPase activity, an important ion transport regulator of alveolar fluid clearance marker, was determined in lung homogenate using the Na^+^/K^+^/ATPase microplate assay kit (orb390725, Biorbyt, Cambridge, United Kingdom). All assays were used according to the manufacturer’s instructions. Haematoxylin and eosin (H&E)-stained lung sections were prepared and evaluated under a standard light microscope by a blinded investigator using the lung injury scoring system as stated by the American Thoracic Society [12]. The two lower lobes of the right lung were removed, weighed and placed in a stove for 7 days at 37°C, after which they were weighed again for calculation of the wet-to-dry weight ratios (Scale: Acculab, Sartorius Group, Bradford, USA). Kidney, liver and heart, wet-to-dry weight ratios were calculated as described for the lung. All parameters and measurements can be found in the supplementary data file (S1 Dataset).

### Statistical analyses

Statistical analyses were performed using R statistical programming software [14] and the ‘car’ [15] and ‘lme4’ [16] packages were used for two-way ANOVA and mixed-effects model analyses. Data was analyzed by two-way ANOVA, with the Tukey post-hoc analysis. A linear mixed-effects model with a random intercept per animal was used to analyze the effect of time (as a continuous variable), age and treatment on blood pressure and static lung compliance measured at hourly intervals, and PaO_2_/FiO_2_ ratios measured at start, mid and end of the mechanical ventilation period. A *p* value less than 0.05 was considered statistically significant.

## Results

**Fig 1:**
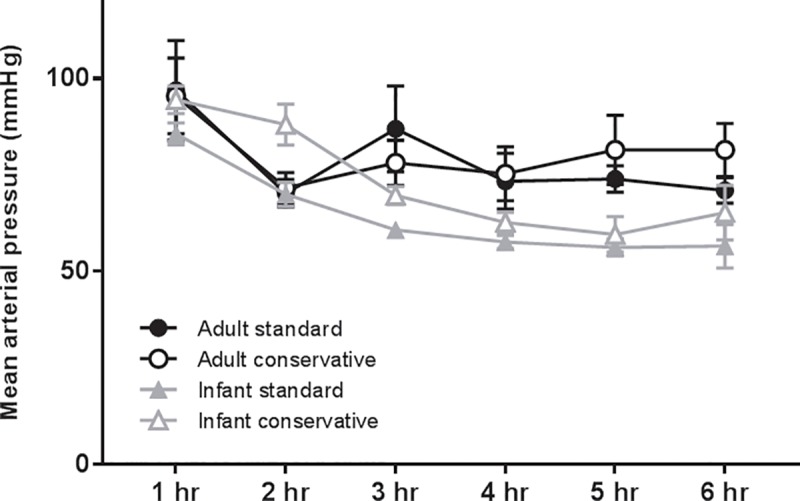
Hemodynamic response is not affected by fluid strategy. The mean arterial blood pressure per experimental group of LPS-inoculated and mechanically ventilated rats. Data are presented as mean + SEM; n = 6–8 animals per group. p = NS.

The use of a conservative fluid strategy was not associated with worsening of the hemodynamic response, as measured by mean arterial blood pressure, during MV in either the adult or infant rats with acute lung injury (p = 0.82; Fig 1). In the infant rats, two animals, one per fluid group, needed intervention for hypotension (either fluid or norepinephrine boluses); in the adult rats, only one rat, in the standard group, needed intervention (Table 1).

**Table 1:** Hemodynamic interventions per experimental group. In all groups (except the adult conservative fluid group), one animal needed interventions for hemodynamic instability. Specified per animal. (no. of fluid boluses (10 ml/kg) or no. noradrenalin boluses, 10 μg/kg)).

Adult–standard fluid (no. animals)		Adult–conservative fluid (no. animals)		Infant–standard fluid (no. animals)		Infant–conservative fluid (no. animals)	
1		0		1		1	
**Fluid**	**Noradrenalin**	**Fluid**	**Noradrenalin**	**Fluid**	**Noradrenalin**	**Fluid**	**Noradrenalin**
3	1	0	0	2	0	0	2

**Fig 2:**
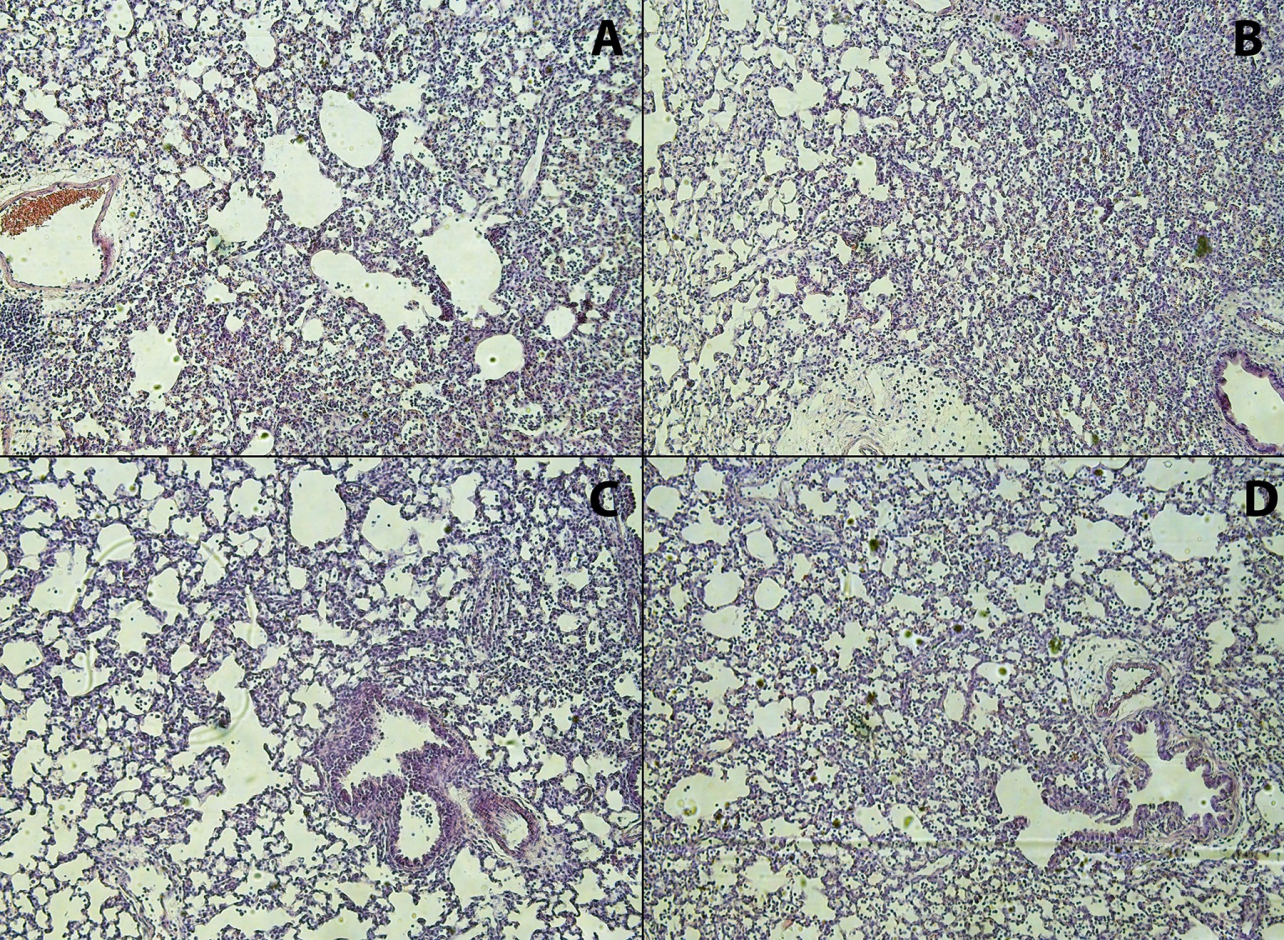
Representative images of lung pathology of each experimental group. A: Adults with standard fluid strategy; B: Adults with conservative fluid strategy; C: Infants with standard fluid strategy; D: Infants with conservative fluid strategy. Magnification: 100X.

Lung pathology showed mean lung injury scores of 0.48 (±0.07) in adult animals with standard fluid, 0.42 (±0.09) in adult animals with conservative fluid, 0.40 (±0.14) in infant animals with standard fluid and 0.46 (±0.13) in infant animals with conservative fluid. No significant differences in these lung injury scores were detected between age or fluid regimen groups (p>0.05). In Fig 2 representative images of the H&E stained lung tissue sections per group are presented.

**Fig 3:**
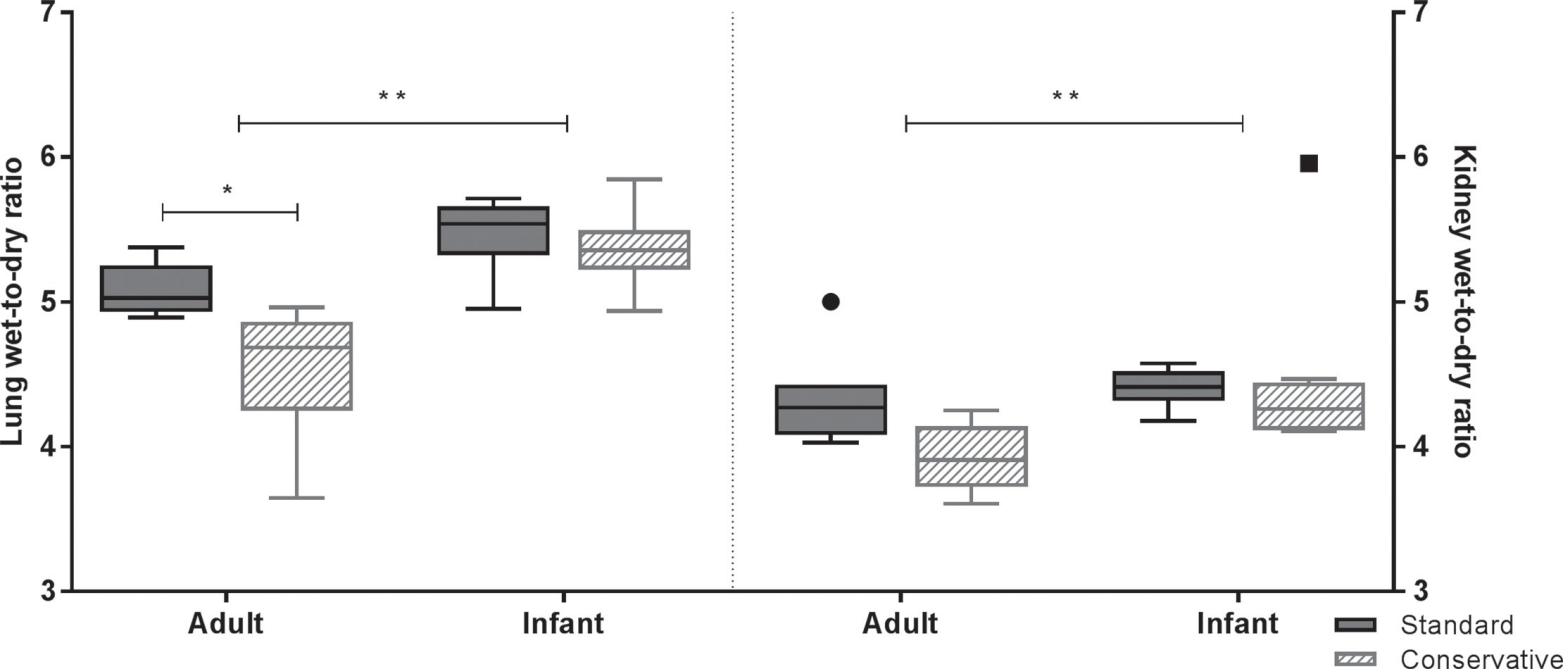
Lung and kidney wet-to-dry ratios. Lung and kidney wet-to-dry weight ratios of each experimental group of LPS-inoculated and mechanically ventilated rats. Data are presented as median + interquartile range [IQR], the whiskers represent 1.5 IQR; n = 6–8 animals per group. *p<0.05, **p<0.01.

In adult rats, a conservative fluid strategy resulted in lower lung wet-to-dry ratios as compared to the standard fluid strategy; yet this was not the case in infants. Apart from the kidney, where a similar (non-significant) trend was seen (p>0.05, Fig 3), wet-to-dry ratios in heart and liver showed no significant differences between the fluid strategies (S1 and S2 Figs). Infant rats had higher total extravascular lung water, as measured by wet-to-dry ratios, in the lungs as compared to adults (p<0.05, Fig 3). This is consistent with previous observations of increased extravascular lung water in children compared to adults [17]. This effect of age was also observed in other organs like the kidney (Fig 3), heart and liver (S1 and S2 Figs), showing overall higher wet-to-dry ratios in infant rats than in adult rats.

**Fig 4:**
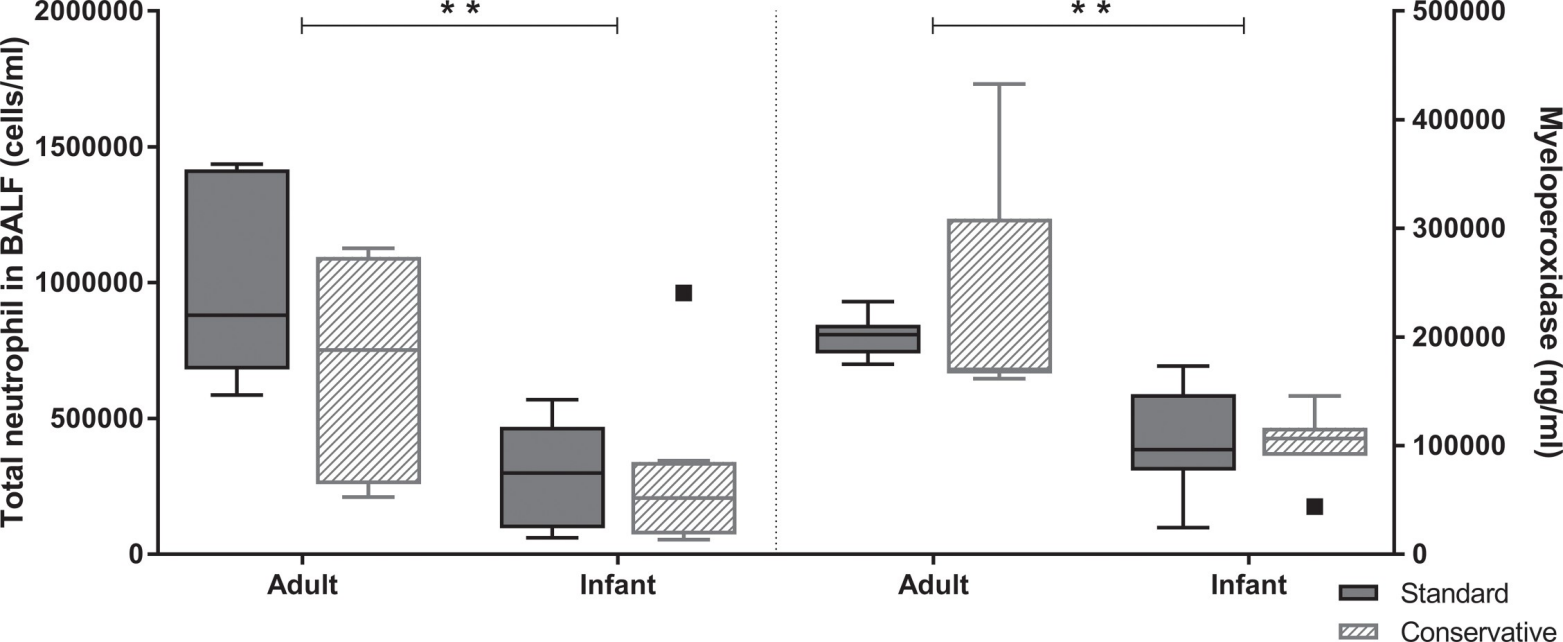
Neutrophil influx and activity is lower in infants than adults. Total neutrophilic cell count in BALF and myeloperoxidase (neutrophil activity marker) in lung homogenate in each experimental group. Data are presented as median + interquartile range [IQR], the whiskers in represent 1.5 IQR; n = 6–8 animals per group. **p<0.01. BALF; bronchoalveolar lavage fluid.

**Fig 5:**
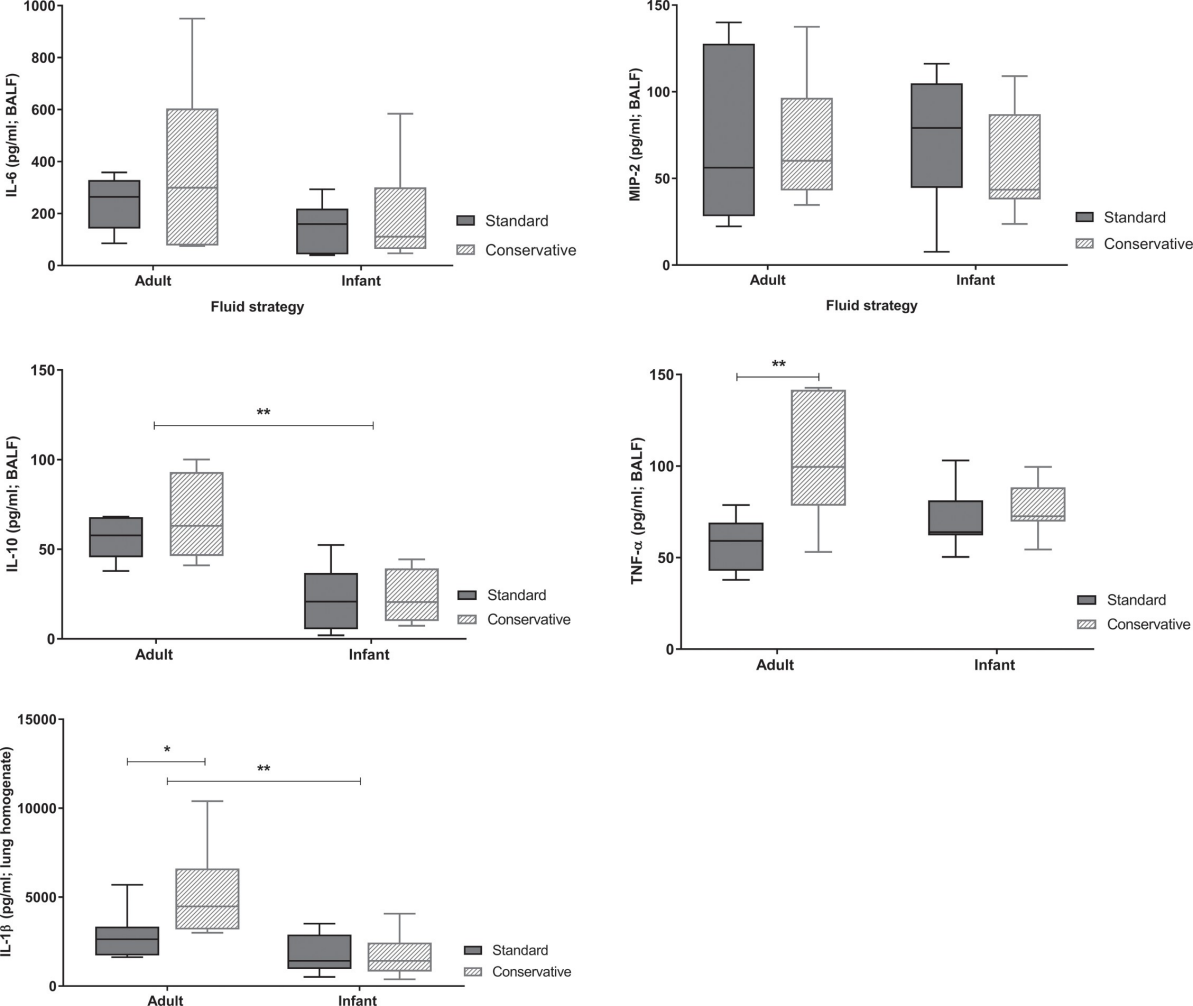
Inflammatory cytokines in the lungs. Inflammatory cytokines IL-6, MIP-2, IL-10 and TNF-α in BALF. IL-1β in lung homogenate. Data are presented as median + interquartile range [IQR], the whiskers in represent 1.5 IQR; n = 6–8 animals per group. p = NS. BALF: bronchoalveolar lavage fluid. *p<0.05, **p<0.01.

The conservative fluid strategy had no effect on neutrophil influx in the lungs or myeloperoxidase activity (Fig 4, p>0.05) compared to the standard strategy group in either adult or infant rats. In contrast, the pro-inflammatory cytokines TNF-α and IL-1β (but not IL-10, IL-6 and MIP-2) were significantly higher in the conservative group compared to the standard group of adult rats. This effect was not present in infant animals (Fig 5). Consistent with previous studies investigating age-dependency of acute lung injury in animals [9, 18], the inflammatory responses were less severe in infant rats as compared to adult rats. Infant rats had less evidence of neutrophilic inflammation in the lungs (BALF total neutrophil counts and MPO in lung homogenate, Fig 4, p < 0.05) in response to LPS and MV as compared to adult animals. Inflammatory IL-10 and IL-1β showed a similar age-dependent effect being lower in infant animals. Nevertheless, the concentrations of the pro-inflammatory cytokines IL-6, MIP-2 and TNF-α in BALF were similar in infant and adult rats (Fig 5, p>0.05).

**Fig 6:**
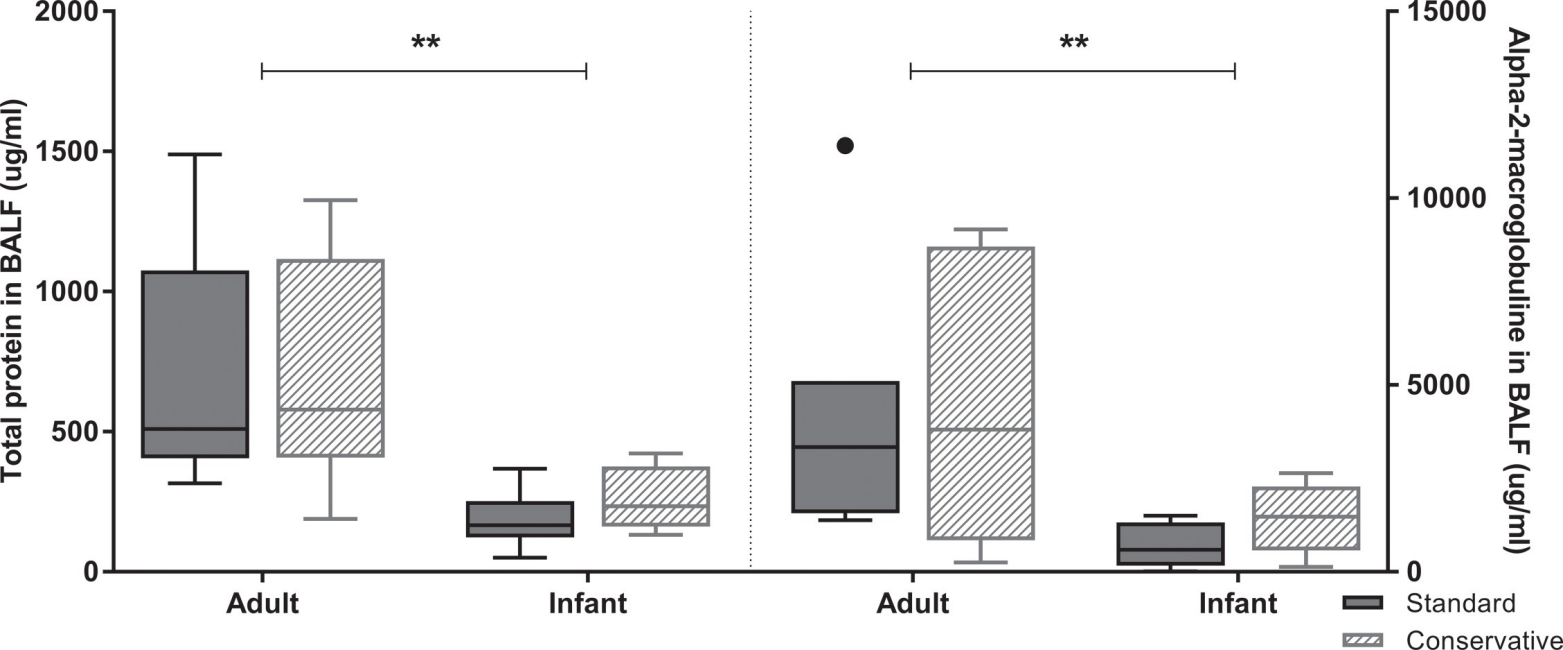
Alveolar permeability is lower in infants than adults. Permeability markers total protein and alpha-2-macroglobulin in BALF in each experimental group. Data are presented as median + interquartile range [IQR], the whiskers in represent 1.5 IQR; n = 6–8 animals per group. **p<0.01. BALF: bronchoalveolar lavage fluid.

**Fig 7:**
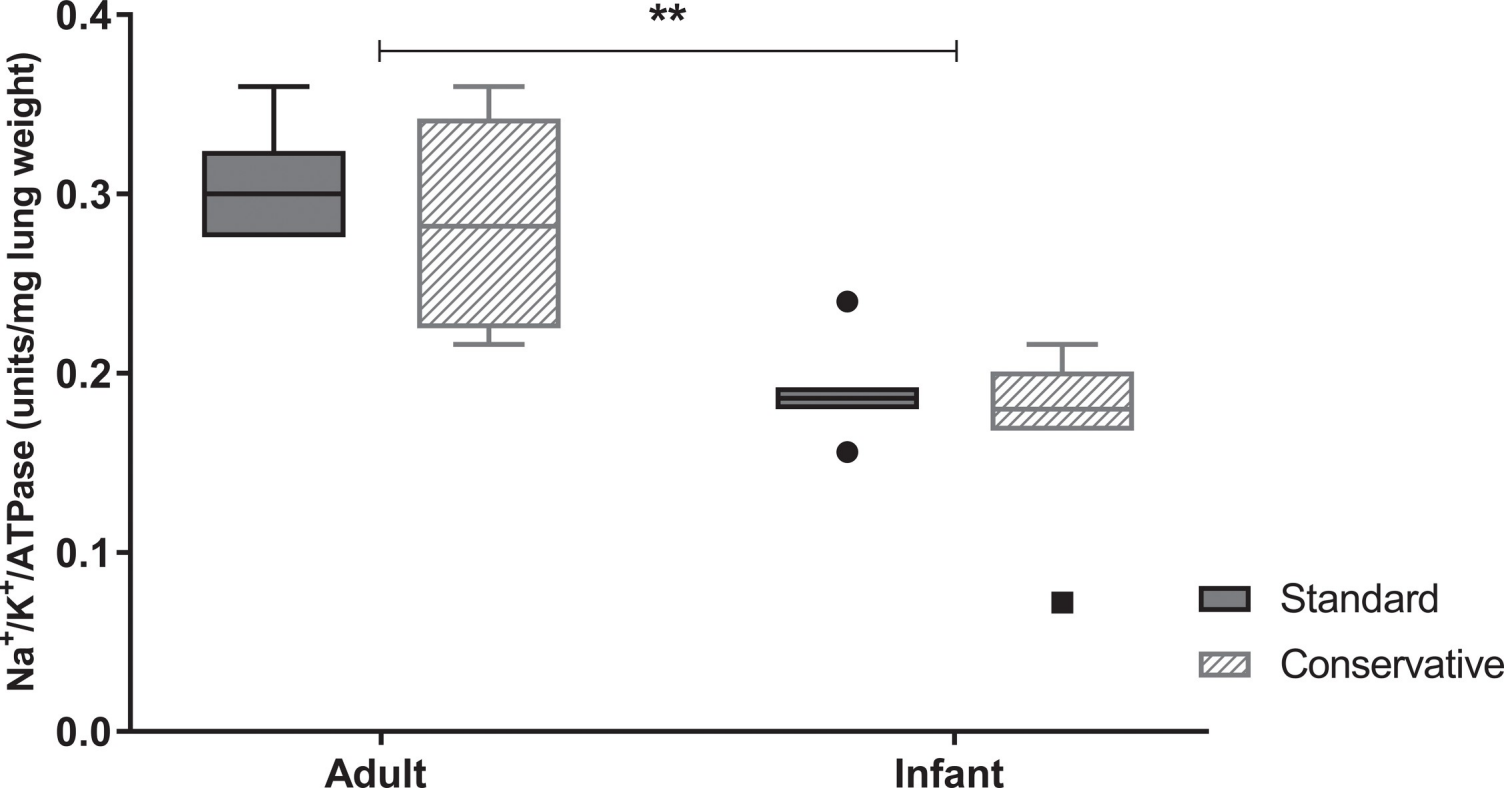
Epithelial cell sodium potassium ATPase pump activity in the lung. Epithelial cell sodium potassium ATPase pump activity (Na^+^/K^+^/ATPase) was assessed in lung homogenate of rats in each experimental group. Data are presented as median + interquartile range [IQR], the whiskers in represent 1.5 IQR; n = 6–8 animals per group.

Similar to the age-dependent effect on inflammation, there was a less severe increase in alveolar permeability as measured by total protein and alpha-2 macroglobulin efflux in BALF in infants as compared to adult rats (Fig 6, p < 0.05). However, we did not see an effect on alveolar permeability of a conservative fluid strategy in either age group. To assess one of the mechanisms of alveolar fluid clearance, epithelial cell sodium potassium ATPase (Na^+^/K^+^/ATPase) pump activity was measured (Fig 7). We found no effect of fluid strategy on Na^+^/K^+^/ATPase activity in either age group tested, yet Na^+^/K^+^/ATPase activity was lower in infants than adults (p<0.05)

**Fig 8:**
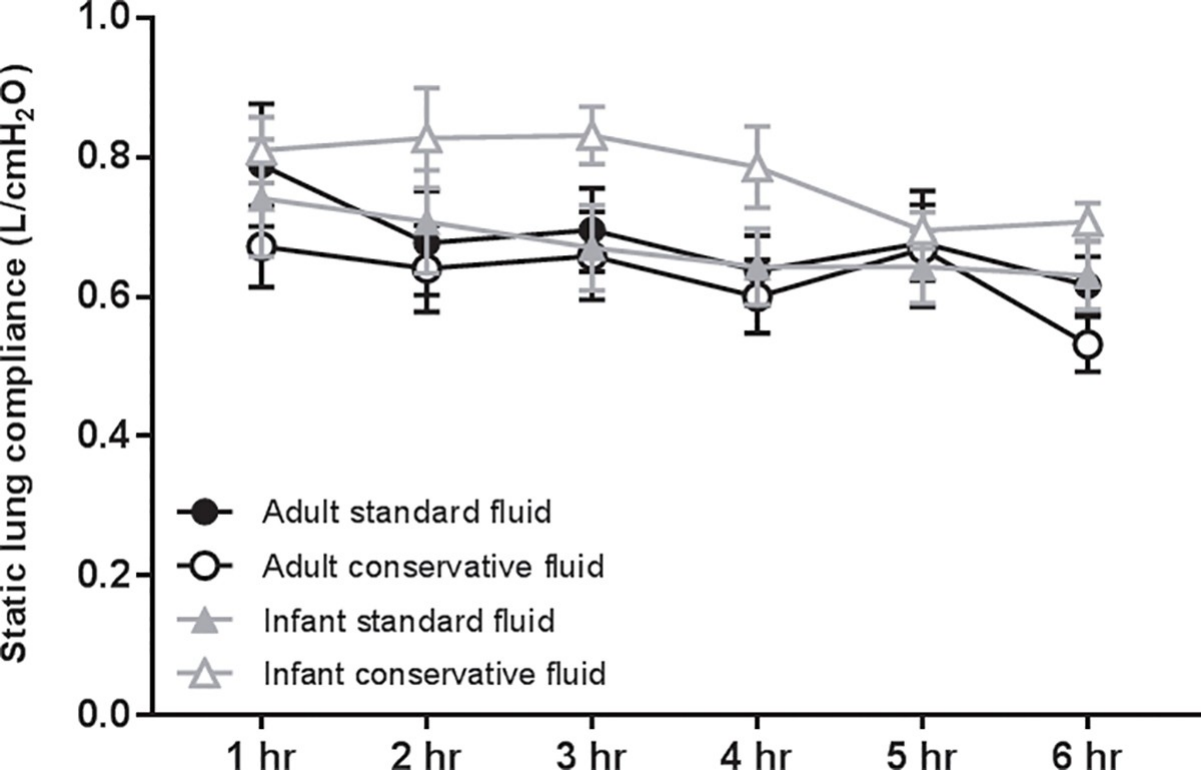
Static lung compliance. Static lung compliance per experimental group of LPS-inoculated and mechanically ventilated rats. Data are presented as mean + SEM; n = 6–8 animals per group. p = 0.89.

**Fig 9:**
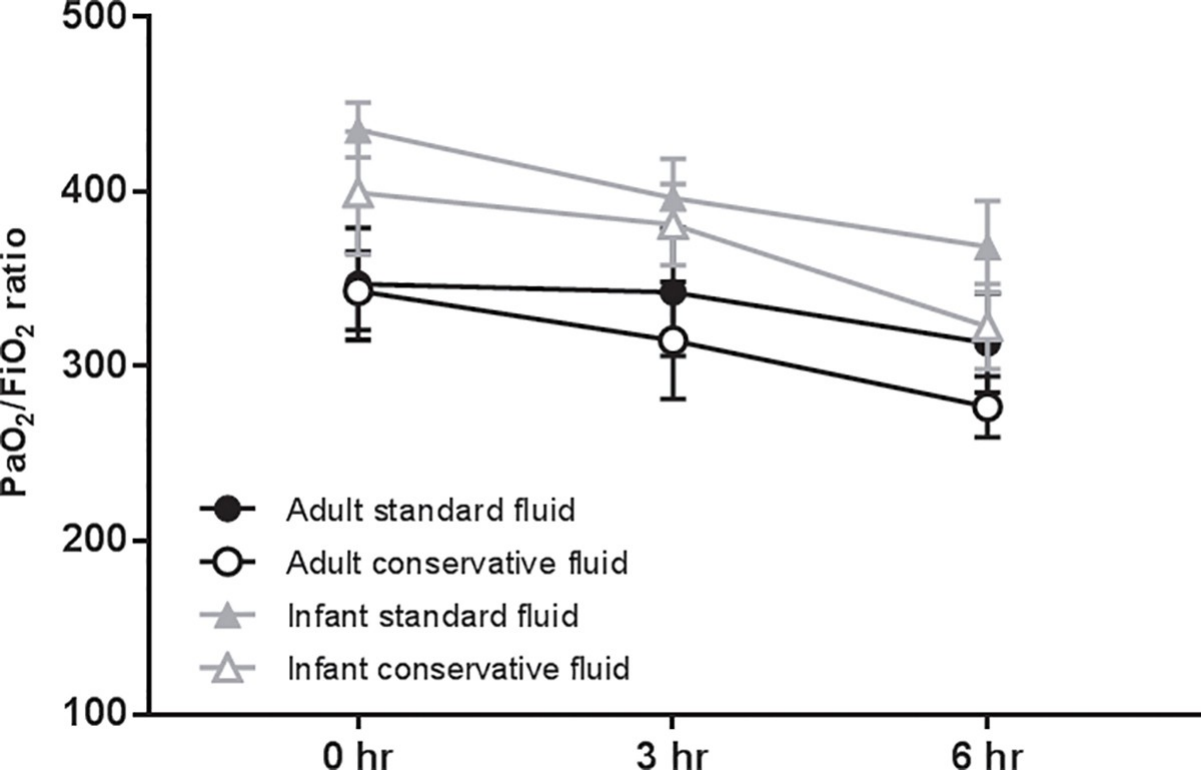
PaO_2_/FiO_2_ ratios. PaO_2_/FiO_2_ ratios at start (T = 0 hr), mid (T = 3 hr) and end (T = 6 hr) of the period of mechanical ventilation per experimental group of LPS-inoculated and ventilated rats. Data are presented as mean + SEM; n = 6–8 animals per group. p = 0.52.

During the short period of MV in this study, a decline in static lung compliance and PaO_2_/FiO_2_ ratios was observed in both age groups, which was unaffected by the fluid regimen (Figs 8 and 9, p = 0.89 for static compliance; p = 0.52 for PaO_2_/FiO_2_ ratios).

## Discussion

In this rat model of acute lung injury induced by intratracheal LPS and MV, a conservative fluid regimen led to diminished extravascular lung water in the lungs. This effect only occurred in adult, not in infant rats.

In experimental animal models, the choice of intravenous fluid maintenance varies widely [9, 19–21]. For example, in rat models rates starting at 1 ml/kg/hour [9] up to 10 ml/kg/hour [11, 21] have been used, and sometimes even with volumes varying between animals within a study [11]. It is an interesting realization, that while evidence of the adverse effects of fluid overload in ARDS patients is accumulating [1, 4], fluid strategies in animal models are not already under a stricter, well-formed policy. While experimental animal models continue to be one of the cornerstones in medical research, translating its results and interpretations into clinical practice is already one of the most challenging tasks in research. Our study shows that the choice of fluid regimen has potential consequences on biophysical markers of tissue edema during acute lung injury in animal models. In adult animals we observed a significant effect on lung wet-to-dry ratios between fluid strategy groups, which is among the most used and significant lung injury markers [12]. In itself fluid strategy did not induce changes in lung permeability or alveolar fluid clearance, suggesting that the increased lung edema in the standard fluid regimen is merely the result of increased extravascular distribution of water in injured, and thus more permeable lungs.

Fluid strategy did have an effect on several markers of inflammation in adult (but not infant) rats. While mechanical stress resulting from heightened hydrostatic pressure in the setting of fluid overload has previously been associated with activation of lung inflammatory responses [5, 6, 22], we found higher concentrations of IL-1β and TNF-α in the conservative fluid group. Considering these outcomes, it becomes clear that conclusions resulting from animal research could be importantly impacted by the choice of fluid strategy. Hence, we advocate that a more uniform, and conservative fluid regimen should be used to ensure comparability among experimental studies.

Age seems to be an important factor in the disease process of ARDS, with children being less susceptible and less severely affected than adults [23, 24]. A similar age-dependent effect has been described in several experimental animal studies of different acute lung injury models [18, 25, 26]. Our study supports this, as the lung inflammatory and permeability responses to equivalent doses of LPS were less severe in infant rats, compared to adult animals. Moreover, we found higher wet-to-dry ratios of all organs (lung, kidney, heart and liver) in infant animals as compared to adults, acknowledging the differences in fluid homeostasis and body water distribution between infants and adults [27]. Interestingly, we found lower Na^+^/K^+^/ATPase activity in infant rats as compared to adults, implying age-differences in alveolar fluid clearance. As previously hypothesized [3], the relatively higher extravascular lung water content under normal conditions in children [17], may make infants and children less prone to develop additional pulmonary edema during ARDS as a consequence of fluid overload. While indeed in our study the relatively high lung wet-to-dry ratios in infant animals were unaffected by restricting their fluid intake, it remains possible that this would be the case in more injured (and thus more permeable) infant lungs.

In ARDS and critically ill patients, fluid overload is considered deleterious on outcome by several potential mechanisms [2, 4, 28]. The major mechanism is considered to be aggravation of interstitial edema in organs such as the lungs and kidneys leading to impaired oxygenation and perfusion [3]. This enhanced tissue edema occurs as a consequence of increased hydrostatic pressure and degradation of the glycocalyx by intravascular fluid challenge [6, 29]. Injured lungs, in which the alveolar capillary barrier has been disrupted, may be more prone to formation of interstitial and intra-alveolar edema during fluid overload. Indeed, in our experimental study, adult animal lungs that were injured by LPS had less extravascular water content upon administration of reduced systemic intravenous fluids. A limitation of our study is that the determinants of extravascular lung water were not assessed, and therefore mechanistic statements are not possible. However, this study was not designed to determine the mechanisms whereby adults and juveniles respond different to fluids. Instead, it focuses on the effect of fluid strategy on important and relevant injury parameters in the setting of experimental acute lung injury.

Given the strong and consistent negative association of fluid overload with clinical outcomes in ARDS and ICU patients in retrospective cohort studies [2, 28, 30–32], it is not remarkable that strategies aimed at limiting fluid overload (fluid restriction and/or diuresis) in the ICU are currently receiving great attention. In the ARDS Network Fluid and Catheters Treatment Trial (FACTT) strict protocolized conservative fluid management led to improved oxygenation and shorter duration of mechanical ventilation and ICU stay [1], as compared to a liberal fluid approach. Importantly, in this trial, patients receiving fluid restriction did not have evidence of impaired (non-pulmonary) organ perfusion. Although animals had a similar hemodynamic tolerance for fluid restriction in our study, we did not find an effect of the conservative fluid strategy on clinical and physiological markers of disease, such as gas exchange and lung compliance. However, our intervention was relatively short, 6 hours, which may not be sufficient time for the development of detectable clinical differences based on the appearance of reduced pulmonary edema. This is also one of the limitations of small animal studies, as it is very difficult to ventilate small animals for longer periods of time. Therefore, it is possible that the lack of changes in gas exchange are due to this short duration.

In conclusion, the choice of fluid strategy in an experimental LPS-induced acute lung injury model of mechanically ventilated rats has a significant effect on at least one of the important and well-recognized lung injury markers. Even though, the impact of fluid strategy seems limited, we advocate the use of a more uniform, conservative, fluid strategy regimen in experimental models of acute lung injury.

## Supporting information

S1 Fig Heart wet-to-dry ratio. Heart wet-to-dry weight ratio of each experimental group of LPS-inoculated and mechanically ventilated rats. Data are presented as median + interquartile range [IQR], the whiskers represent 1.5 IQR; n = 6–8 animals per group. **p<0.01.
(PDF)

S2 Fig Liver wet-to-dry weight ratio. Liver wet-to-dry weight ratio of each experimental group of LPS-inoculated and mechanically ventilated rats. Data are presented as median + interquartile range [IQR], the whiskers represent 1.5 IQR; n = 6–8 animals per group. **p<0.01.
(PDF)

S1 Dataset Raw dataset of all parameters and measurements obtained during this research. (XLSX)
