# Supplementary figures and images for: Fluid restriction reduces pulmonary edema in a model of acute lung injury in mechanically ventilated rats
Source: PLoS One. 2019 Jan 17;14(1):e0210172. doi: 10.1371/journal.pone.0210172 (PMC6336323; doi:10.1371/journal.pone.0210172)

## Supporting Information

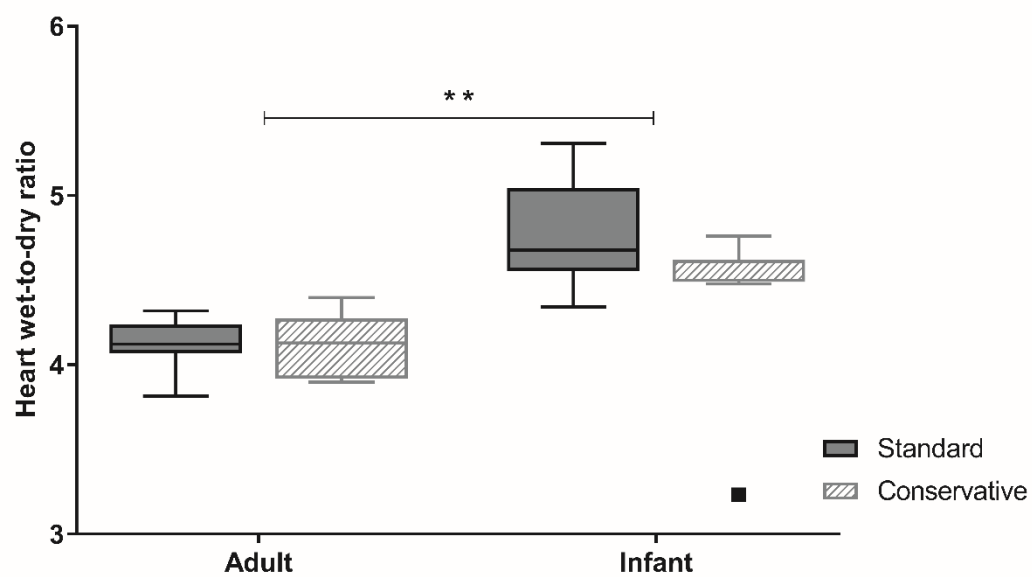

Supplement: S1 Fig — Heart wet-to-dry weight ratio of each experimental group of LPS-inoculated and mechanically ventilated rats. Data are presented as median + interquartile range [IQR], the whiskers represent 1.5 IQR; n = 6–8 animals per group. **p<0.01. (PDF) [file pone.0210172.s001.pdf]

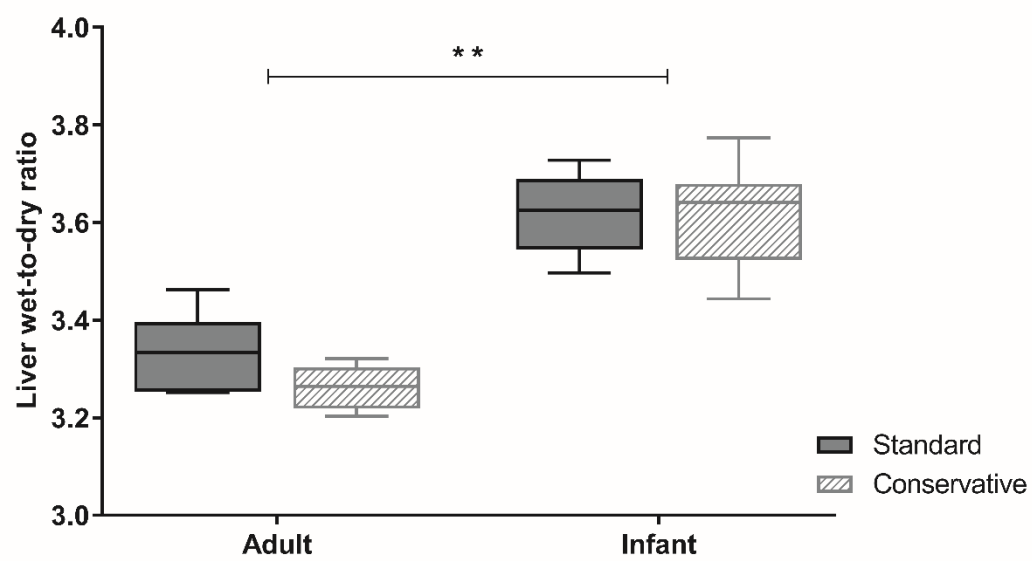

Supplement: S2 Fig — Liver wet-to-dry weight ratio of each experimental group of LPS-inoculated and mechanically ventilated rats. Data are presented as median + interquartile range [IQR], the whiskers represent 1.5 IQR; n = 6–8 animals per group. **p<0.01. (PDF) [file pone.0210172.s002.pdf]
